# Second Time's the Charm? Assessing the Sensitivity and Yield of Inpatient Diagnostic Algorithms for Pulmonary Tuberculosis in a Low-Prevalence Setting

**DOI:** 10.1093/ofid/ofae253

**Published:** 2024-05-03

**Authors:** Caitlin M Dugdale, Kimon C Zachary, Dustin S McEvoy, John A Branda, Amy Courtney, Rebecca Craig, Alexandra Doms, Lindsay Germaine, Chloe V Green, Eren Gulbas, David C Hooper, Rocio M Hurtado, Emily P Hyle, Michelle S Jerry, Jacob E Lazarus, Molly Paras, Sarah E Turbett, Erica S Shenoy

**Affiliations:** Medical Practice Evaluation Center, Department of Medicine, Massachusetts General Hospital, Boston, Massachusetts, USA; Division of Infectious Diseases, Department of Medicine, Massachusetts General Hospital, Boston, Massachusetts, USA; Harvard Medical School, Boston, Massachusetts, USA; Division of Infectious Diseases, Department of Medicine, Massachusetts General Hospital, Boston, Massachusetts, USA; Harvard Medical School, Boston, Massachusetts, USA; Infection Control, Massachusetts General Hospital and Mass General Brigham, Boston, Massachusetts, USA; Clinical Informatics, Mass General Brigham, Boston, Massachusetts, USA; Harvard Medical School, Boston, Massachusetts, USA; Department of Pathology, Massachusetts General Hospital, Boston, Massachusetts, USA; Infection Control, Massachusetts General Hospital and Mass General Brigham, Boston, Massachusetts, USA; Infection Control, Massachusetts General Hospital and Mass General Brigham, Boston, Massachusetts, USA; Department of Medicine, Massachusetts General Hospital, Boston, Massachusetts, USA; Clinical Informatics, Mass General Brigham, Boston, Massachusetts, USA; Infection Control, Massachusetts General Hospital and Mass General Brigham, Boston, Massachusetts, USA; Medical Practice Evaluation Center, Department of Medicine, Massachusetts General Hospital, Boston, Massachusetts, USA; Division of Infectious Diseases, Department of Medicine, Massachusetts General Hospital, Boston, Massachusetts, USA; Harvard Medical School, Boston, Massachusetts, USA; Infection Control, Massachusetts General Hospital and Mass General Brigham, Boston, Massachusetts, USA; Division of Infectious Diseases, Department of Medicine, Massachusetts General Hospital, Boston, Massachusetts, USA; Harvard Medical School, Boston, Massachusetts, USA; Global Health Committee, Boston, Massachusetts, USA; Medical Practice Evaluation Center, Department of Medicine, Massachusetts General Hospital, Boston, Massachusetts, USA; Division of Infectious Diseases, Department of Medicine, Massachusetts General Hospital, Boston, Massachusetts, USA; Harvard Medical School, Boston, Massachusetts, USA; Infection Control, Massachusetts General Hospital and Mass General Brigham, Boston, Massachusetts, USA; Division of Infectious Diseases, Department of Medicine, Massachusetts General Hospital, Boston, Massachusetts, USA; Harvard Medical School, Boston, Massachusetts, USA; Division of Infectious Diseases, Department of Medicine, Massachusetts General Hospital, Boston, Massachusetts, USA; Harvard Medical School, Boston, Massachusetts, USA; Division of Infectious Diseases, Department of Medicine, Massachusetts General Hospital, Boston, Massachusetts, USA; Harvard Medical School, Boston, Massachusetts, USA; Department of Pathology, Massachusetts General Hospital, Boston, Massachusetts, USA; Department of Medicine, Massachusetts General Hospital, Boston, Massachusetts, USA; Division of Infectious Diseases, Department of Medicine, Massachusetts General Hospital, Boston, Massachusetts, USA; Harvard Medical School, Boston, Massachusetts, USA; Infection Control, Massachusetts General Hospital and Mass General Brigham, Boston, Massachusetts, USA

**Keywords:** acid-fast bacilli (AFB) smear, *Mycobacterium tuberculosis*, Xpert MTB/RIF NAAT

## Abstract

**Background:**

For persons with suspected pulmonary tuberculosis, the guidelines of the Centers for Disease Control and Prevention recommend collecting 3 respiratory specimens 8 to 24 hours apart for acid-fast bacilli (AFB) smear and culture, in addition to 1 nucleic acid amplification test (NAAT). However, data supporting this approach are limited. Our objective was to estimate the performance of 1, 2, or 3 AFB smears with or without NAATs to detect pulmonary tuberculosis in a low-prevalence setting.

**Methods:**

We conducted a retrospective study of hospitalized persons at 8 Massachusetts acute care facilities who underwent mycobacterial culture on 1 or more respiratory specimens between July 2016 and December 2022. We evaluated percentage positivity and yield on serial AFB smears and NAATs among people with growth of *Mycobacterium tuberculosis* on mycobacterial cultures.

**Results:**

Among 104 participants with culture-confirmed pulmonary tuberculosis, the first AFB smear was positive in 41 cases (39%). A second AFB smear was positive in 11 (22%) of the 49 cases in which it was performed. No third AFB smears were positive following 2 initial negative smears. Of 52 smear-negative cases, 36 had a NAAT performed, leading to 23 additional diagnoses. Overall sensitivity to detect tuberculosis prior to culture positivity was higher in any strategy involving 1 or 2 NAATs (74%–79%), even without AFB smears, as compared with 3 smears alone (60%).

**Conclusions:**

Tuberculosis diagnostic testing with 2 AFB smears offered the same yield as 3 AFB smears while potentially reducing laboratory burden and duration of airborne infection isolation. Use of 1 or 2 NAATs increased sensitivity to detect culture-positive pulmonary tuberculosis when added to AFB smear–based diagnostic testing alone.

## Graphical Abstract

**Graphical Abstract:**
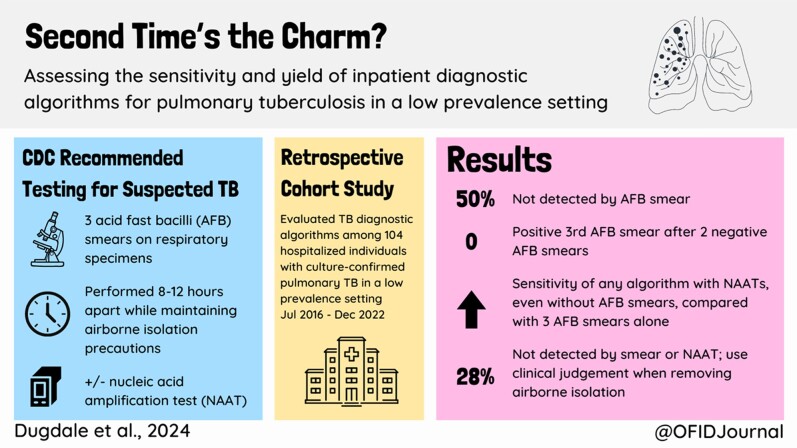

## Teaser

Centers for Disease Control and Prevention guidelines recommend 3 acid-fast bacilli smears and 1 nucleic acid amplification test when pulmonary tuberculosis is suspected, but data supporting this practice are limited. We report the performance of diagnostic algorithms consisting of combinations of acid-fast bacilli smears and nucleic acid amplification tests in a low-prevalence setting.

In 2022, 8300 new cases of *Mycobacterium tuberculosis* were reported in the United States, and the risk of health care–associated transmission of *M tuberculosis* remains a concern [1, 2]. When persons with a clinical syndrome consistent with possible pulmonary tuberculosis are identified in health care settings, airborne precautions and placement of the person in an airborne infection isolation room should be initiated promptly to reduce the risks of transmission to other patients, health care personnel, and visitors [3]. However, when active tuberculosis is no longer suspected after a negative diagnostic workup, prompt discontinuation of isolation is important to reduce facility length of stay [4] and preserve hospital capacity [4], as airborne infection isolation rooms are a limited resource [5].

Among persons for whom there is a concern for tuberculosis, guidelines endorsed by the Centers for Disease Control and Prevention recommend collecting 3 consecutive respiratory specimens 8 to 24 hours apart for acid-fast bacilli (AFB) smear and mycobacterial culture, as well as at least 1 nucleic acid amplification test (NAAT) [2, 6, 7]. In 2015, the US Food and Drug Administration updated device labeling for the Xpert MTB/RIF assay (Cepheid), a NAAT, to indicate that the use of NAATs on 1 or 2 sputum specimens is a reasonable alternative to assessment of serial AFB smears [8]. Given the higher reported sensitivity and specificity of a NAAT as compared with an AFB smear [7], some health care facilities now utilize 2 consecutive NAATs to guide discontinuation of airborne precautions in lieu of 3 AFB smears [9].

Use of NAATs for tuberculosis diagnostic testing has been shown to reduce person time in airborne isolation [10–13], duration of hospitalization [4], and costs [4] as compared with AFB smears alone, as well as delays in the initiation of tuberculosis treatment [14]. The performance of AFB smears is resource intensive for microbiology laboratories as compared with NAATs, requiring manual processing and microscopy review by trained technicians 7 days a week. AFB smear microscopy is not feasible for many hospital laboratories that are already facing unprecedented staffing challenges [15], so these specimens are often sent to reference laboratories, potentially contributing to delays in care. Conversely, NAAT platforms, which are increasingly available in clinical laboratories [16], are less labor intensive but have higher up-front costs than microscopy [11], limiting their availability in some health care settings. AFB smears and culture provide value beyond NAATs, including detection of nontuberculous mycobacteria and estimation of the overall bacillary burden, which inform the duration of infectiousness of individuals with tuberculosis, the contact investigations, and the treatment decisions [17]. Regardless of the tuberculosis diagnostic approach, removing airborne isolation prematurely in the setting of active disease can lead to potential health care–associated transmission and resource-intensive exposure investigations, which may require additional screening of exposed patients, health care personnel, and visitors [3, 17]. Given these trade-offs, we evaluated the sensitivity and yield of various AFB smear– and NAAT-based diagnostic algorithms for pulmonary tuberculosis among hospitalized persons in a low-prevalence setting to inform guidelines regarding optimal tuberculosis diagnostic approaches.

## METHODS

We conducted a retrospective study of persons who were hospitalized at any of 8 Mass General Brigham acute care facilities in Massachusetts (supplementary appendix) and had growth of *M tuberculosis* complex in 1 or more mycobacterial cultures from respiratory specimens collected between July 2016 and December 2022. We included the results of all AFB smears and NAATs performed on qualifying respiratory specimens collected at the Mass General Brigham facilities, including the results of some tests that were performed at the Massachusetts State Laboratory.

Results of all mycobacterial cultures, AFB smears, and NAATs were extracted from the electronic health record data warehouse (Epic Inc). We extracted data—demographic, epidemiologic (eg, known risk factors for tuberculosis exposure [7]), clinical, and radiographic—from the electronic health record using a standardized data collection form.

In the primary analysis, we evaluated the sensitivity of a variety of *M tuberculosis* diagnostic algorithms consisting of combinations of smears and NAATs on respiratory specimens as compared with the gold standard of a positive mycobacterial culture. For each diagnostic algorithm, we evaluated all smears and NAATs performed within a 7-day period, which we defined as a “testing episode.” Participants were considered eligible to contribute to the calculation of algorithm sensitivity if they had at least as many smears and NAATs performed during the testing episode as required by the algorithm. However, not all patients had sufficient tests performed to be included in the sensitivity evaluation of all algorithms. Therefore, we calculated the observed yield of each testing algorithm, defined as the proportion of all individuals with tuberculosis in our study, irrespective of how many tests each person had performed, who would have been detected by each testing algorithm. In the yield calculation, an individual was counted as positive in all algorithms for which they had any positive algorithm component (eg, if they had a first AFB smear that was positive, they were counted as positive in all algorithms with at least 1 AFB smear). We calculated Clopper-Pearson binomial confidence intervals for algorithm sensitivity and yield as compared with the gold standard of a positive mycobacterial culture from a respiratory specimen.

In secondary analyses, we evaluated the sensitivity of AFB smears and NAATs by specimen type. For testing episodes in which the participants were diagnosed with tuberculosis, we compared the performance of sputum specimens (spontaneous or induced) and tracheal aspirates vs that of bronchoscopic specimens (bronchoalveolar lavage and bronchial washing) using a chi-square test. *P* < .05 was considered statistically significant. All statistical analyses were performed with Stata version 17. The study was approved by the Mass General Brigham Institutional Review Board (2012P002359).

## RESULTS

There were 104 participants with a respiratory mycobacterial culture positive for *M tuberculosis* during the study period. These participants with pulmonary tuberculosis underwent 312 mycobacterial cultures during the initial testing episode in which they were diagnosed, with a median 3 mycobacterial cultures per participant (IQR, 3–4; range, 1–8). The majority of cultures were from sputum or tracheal aspirates (256/312, 82%) with the remainder from bronchoscopic specimens (56/312, 18%). Of the 104 participants, 85 (82%) had at least 1 NAAT performed. Among all participants, 89 (86%) had a positive culture on their first specimen; 12 (12%) had a first positive culture on their second specimen; and 3 (3%) had a first positive culture on their third specimen, all 3 of whom were from highly tuberculosis-endemic geographic areas and had abnormal chest imaging findings (Supplementary Appendix Table 1).

The median age of participants was 52 years (IQR, 28–69), and 60 of 104 (58%) were of male legal sex (Table 1). In 11 (11%) individuals with tuberculosis, no epidemiologic risk factor for tuberculosis was documented. Common presenting symptoms included cough (61%), weight loss (53%), fever (46%), and night sweats (27%); however, 13% of individuals did not have any classic symptoms of tuberculosis recognized at presentation and largely underwent testing due to incidental radiographic findings. Chest radiograph was performed in 97 (93%) individuals, among whom 24 (25%) had a cavitary lesion or other radiographic finding interpreted as specifically concerning for tuberculosis; 9 (9%) had normal chest radiograph results. Of the 104 participants, computed tomography of the chest was performed in 95 (91%) individuals, among whom 59 (62%) had a cavitary lesion or other finding interpreted as concerning for tuberculosis.

**Table 1.** Characteristics of Hospitalized People Diagnosed With Pulmonary Tuberculosis in the Mass General Brigham System, 2016–2022 (N = 104)

Characteristic	No. (%)^a^
Age, y, median (IQR)	52 (28–69)
Male	60 (58)
Presence of identified risk factor for active TB	
Prior residence in or travel to an endemic country for ≥1 mo	84 (81)
History of incarceration	4 (4)
Prior residence in a homeless shelter	3 (3)
History of known close contact with someone with active TB	7 (7)
History of latent TB^b^	24 (23)
No epidemiologic risk factor identified	11 (11)
Medical history at the time of TB diagnosis^c^	
Immunocompromised^d^	20 (19)
HIV infection	6 (6)
Underweight: body mass index <18.5	8 (8)
Diabetes mellitus	17 (16)
Chronic kidney disease stage ≥4	3 (3)
Chronic obstructive pulmonary disease	5 (5)
Active tobacco smoking	8 (8)
Prior gastrectomy or gastric bypass	2 (2)
Cirrhosis	2 (2)
None of the above	63 (61)
Classic TB symptoms identified at presentation	
Cough	63 (61)
Hoarseness	5 (5)
Hemoptysis	15 (14)
Fever	48 (46)
Night sweats	28 (27)
Weight loss	55 (53)
None of the above	14 (13)
Chest radiograph findings	
Not performed^e^	7 (7)
Cavitary lesions	12 (12)
Other findings described as suspicious for active TB	12 (12)
Other findings not described as suspicious for active TB	64 (62)
No lung, pleura, or lymph node abnormalities	9 (9)
Chest CT findings	
Not performed^e^	9 (9)
Cavitary lesions	28 (27)
Other findings described as suspicious for TB	31 (30)
Other findings not described as suspicious for TB	36 (35)
No lung, pleura, or lymph node abnormalities	0 (0)

Abbreviations: IQR, interquartile range; TB, tuberculosis; CT, computed tomography.

^a^Participants may be counted in multiple categories for each section.

^b^History of latent TB was determined by the person's report of prior preventative therapy for TB or by a prior positive tuberculin skin test or interferon gamma release assay result before the index presentation with active TB infection.

^c^These medical history elements were collected because they are associated with progression to active TB.

^d^Mass General Brigham criteria for an immunocompromised state can be found in the supplementary appendix.

^e^Chest CT was performed in 6 of the 7 individuals who did not have a chest radiograph performed: 1 did not have chest imaging at the time of TB evaluation as CT had been performed during outpatient evaluation 1 month prior.

**Figure 1.**
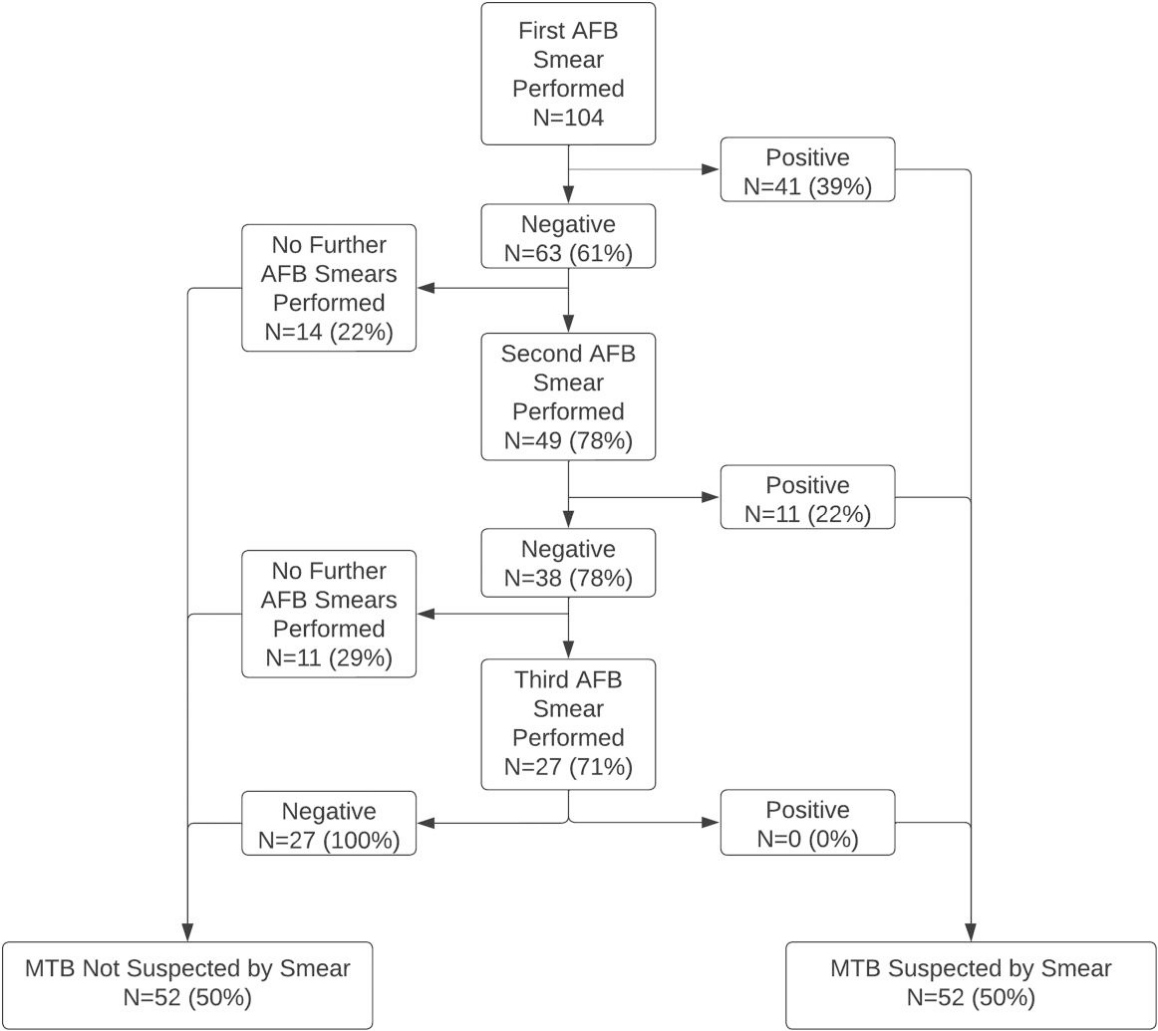
Results of acid-fast bacilli (AFB) smears from respiratory samples among hospitalized persons with confirmed pulmonary tuberculosis as defined by a positive mycobacterial culture. Percentages are provided with the denominator being the number of specimens from the prior step in the cascade. MTB, *Mycobacterium tuberculosis*.

Of the 104 participants with tuberculosis, 41 (39%) had a first AFB smear that was positive (Figure 1). Among the 63 participants with an initial negative smear, 49 (78%) had a second AFB smear performed, which was positive in 11 cases (22%). There were 38 participants with negative first and second smears, of whom 27 (71%) underwent third smears, none of which were positive. In total, 52 (50%) individuals with pulmonary tuberculosis had at least 1 positive AFB smear.

**Figure 2.**
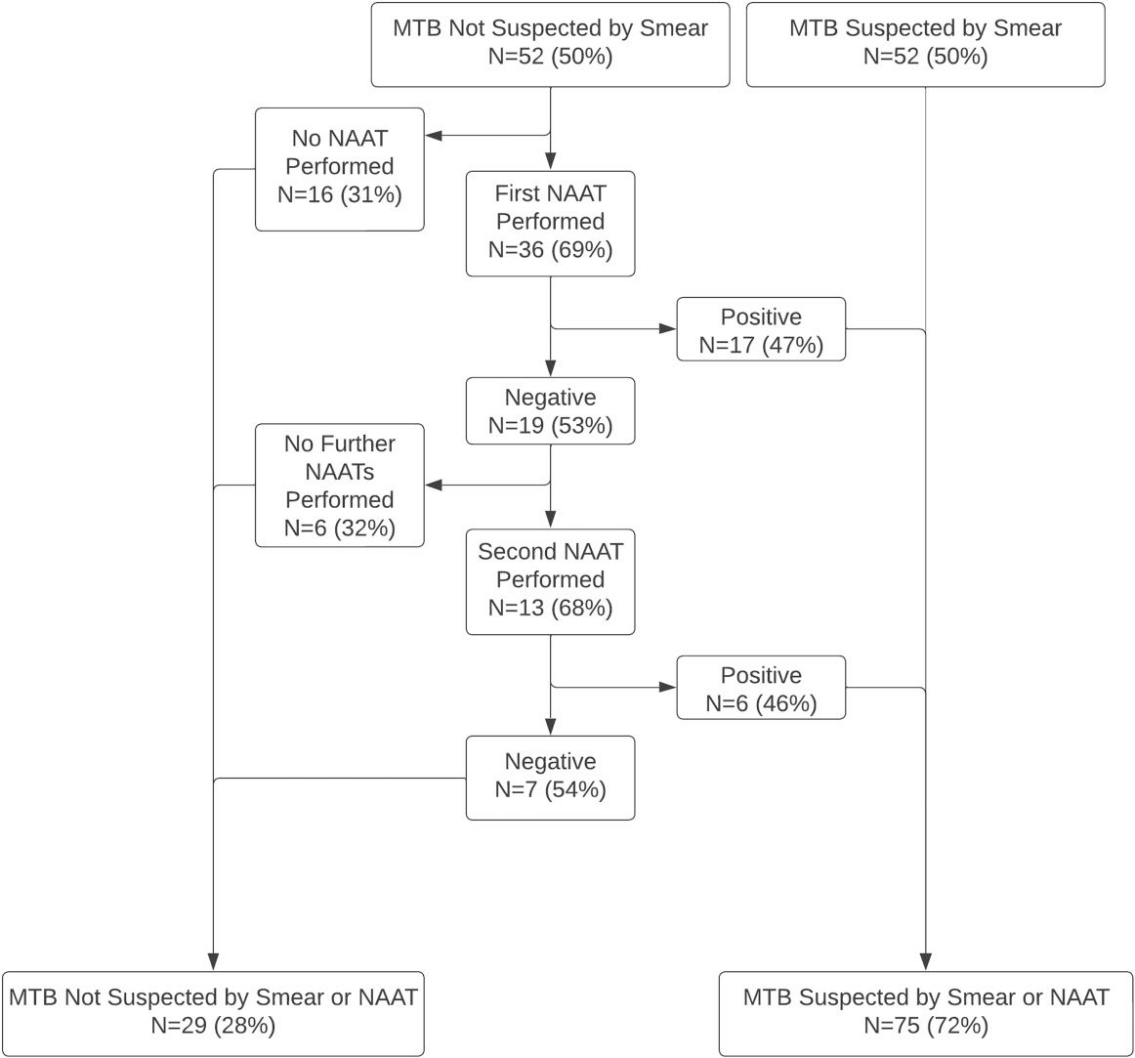
Nucleic acid amplification test (NAAT) results from respiratory samples among hospitalized persons with confirmed pulmonary tuberculosis as defined by a positive mycobacterial culture who had at least 1 negative acid-fast bacilli smear. Percentages are provided with the denominator being the number of specimens from the prior step in the cascade. MTB, *Mycobacterium tuberculosis*.

Among the 52 (50%) of participants with negative smears, 36 (69%) had at least 1 NAAT performed (Figure 2), of whom 17 (47%) tested positive for *M tuberculosis*. Of the 19 participants with a first negative NAAT result, 13 (68%) underwent a second NAAT, and 6 (46%) returned positive. Ultimately, 23 of 52 (44%) participants with all negative smears had a positive NAAT result, leading to a diagnosis of pulmonary tuberculosis. Of the 104 cases of confirmed pulmonary tuberculosis, 29 (28%) were not detected by smear or NAAT during the initial diagnostic evaluation.

The lowest overall sensitivity to detect *M tuberculosis* prior to growth in culture was from the “1 AFB smear” algorithm, with only 41 of 104 participants (39%; 95% CI, 30%–49%) deemed positive by this approach (Table 2). For persons with 2 or 3 smears, observed algorithm sensitivity increased to 54% (44/82; 95% CI, 43%–65%) and 60% (40/67; 95% CI, 47%–72%), respectively. However, the observed yield of 2 and 3 smears was the same (52/104 [50%]; 95% CI, 40%–60%), as no additional participants were detected on a third smear. Adding a NAAT increased the sensitivity to detect tuberculosis beyond any of the smear-based strategies. Of 85 participants, 64 (75%; 95% CI, 65%–84%) had a positive result on the first NAAT. All of the approaches that included at least 1 NAAT led to higher sensitivity than that achieved by smears alone, at 74% to 79%.

**Table 2.** Observed Characteristics of *Mycobacterium tuberculosis* Diagnostic Algorithms in a Low-Prevalence Setting

No. AFB Smears	No. Positive by Algorithm	No. Eligible With MTB by Culture^a^	Observed Sensitivity (95% CI), %^b^	Observed Yield (95% CI), %^c^
AFB smears + 0 NAATs				
1	41	104	39 (30–49)	39 (30–49)
2	44	82	54 (43–65)	50 (40–60)
3	40	67	60 (47–72)	50 (40–60)
AFB smears + 1 NAAT				
0	64	85	75 (65–84)	62 (51–71)
1	64	85	75 (65–84)	63 (53–73)
2	51	68	75 (63–85)	66 (56–75)
3	44	57	77 (64–87)	66 (56–75)
AFB smears + 2 NAATs				
0	22	29	76 (56–90)	68 (58–77)
1	22	29	76 (56–90)	70 (60–79)
2	22	28	79 (59–92)	72 (62–80)
3	14	19	74 (49–91)	72 (62–80)

Abbreviations: AFB, acid-fast bacteria; MTB, *Mycobacterium tuberculosis*; NAAT, nucleic acid amplification test.

^a^Eligibility was determined by having a sufficient number of AFB smears and NAATs performed to assess the characteristics of the algorithm. All included participants had at least 1 AFB smear.

^b^Sensitivity was calculated by dividing the number positive by the algorithm by the number eligible with MTB by culture. The denominator fluctuates, as not all individuals in this real-world retrospective study received sufficient testing to be evaluated in every algorithm.

^c^Observed yield was defined as the proportion of all individuals with culture-positive tuberculosis in the study (104 total) who would have been detected by each algorithm evaluated. In this approach, individuals who were positive by a less intensive testing algorithm (eg, 1 AFB smear) were still counted as positive on more intensive testing algorithms that included that test component (eg, 2 and 3 AFB smears), even if they did not undergo the more intensive testing. Here, for all but the “1 AFB smear” and “1 NAAT ± 1 AFB smear” approaches, the estimate presented reflects a lower bound of the yield of a given testing algorithm, as only a proportion of participants underwent the more intensive testing; that is, most participants in the study did not have 3 AFB smears + 2 NAATs to evaluate the absolute yield of that completed tested algorithm.

Among the 104 participants with at least 1 positive culture, 245 of 312 mycobacterial cultures (79%) obtained during the initial testing episode were positive for *M tuberculosis* (Table 3). Of the 104 participants, 11 also had growth of a nontuberculous mycobacterium in a culture obtained during the initial testing episode (Supplementary Appendix Table 2). The proportion of cultures obtained that returned positive during the initial testing episode did not differ markedly by type of respiratory specimen. There was no significant difference in NAAT positivity by respiratory specimen type among culture-positive specimens (*P* > .05). NAATs from any specimen type were more likely to be positive when the specimen was positive upon AFB smear (57/57, 100%) than negative (26/42, 62%; *P* < .01).

**Table 3.** Comparison by Specimen Type of the Sensitivity of AFB Smear and NAAT Among All Tests Performed During a Testing Episode^a^ in Which Tuberculosis Was Diagnosed

	Bronchoscopic Specimen		Sputum/Tracheal Aspirate		
Test Type	No.	% (95% CI)	No.	% (95% CI)	*P* Value
Mycobacterial culture positivity: among all specimens	40/56	71 (58–83)	205/256	80 (75–85)	.15
AFB smear microscopy: among all culture-positive specimens	15/40	38 (23–54)	118/205	58 (50–64)	.02
NAAT: among all culture-positive specimens	18/24	75 (53–90)	65/75	87 (77–93)	.18
Smear positive	11/11	100 (72–100)	46/46	100 (92–100)	>.99
Smear negative	7/13	54 (25–81)	19/29	65 (46–82)	.47

	No Sputum Specimen Available Prior to Bronchoscopic Sampling		Sputum Specimen Available Prior to Bronchoscopic Sampling		
	No.	% (95% CI)	No.	% (95% CI)	
AFB smear microscopy: among culture-positive bronchoscopic specimens	7/26	27 (12–48)	8/14	57 (29–82)	.09

Abbreviations: AFB, acid-fast bacilli; NAAT, nucleic acid amplification test.

^a^A “testing episode” was defined as the window within 7 days of the initial specimen in a series being collected with any specimen in that series eventually having a mycobacterial culture positive for *Mycobacterium tuberculosis*.

Among culture-positive specimens obtained during the testing episode, AFB smears were more often positive from sputum and tracheal aspirate specimens (118/205, 58%) than from bronchoscopic specimens (15/40, 38%; *P* = .02). However, there was a trend toward higher bronchoscopic specimen smear positivity when sputum specimens were available prior to bronchoscopic sampling than when they were not (57% vs 27%, *P* = .09), which may speak to higher organism burden among patients who could produce sputum. In 14 of 104 participants (13%), the first AFB smear or NAAT to return positive was from a bronchoscopic specimen. For 9 (64%) who were diagnosed by bronchoscopy, sputum could not be produced, even with attempted induction by a respiratory therapist, prompting the need for bronchoscopy for diagnosis. Eight had a sputum specimen collected within 24 hours after bronchoscopy, of whom 1 had the tuberculosis diagnosis made on the postbronchoscopy specimen (Supplementary Appendix Table 3).

## DISCUSSION

Over a 5-year period in a low-prevalence setting, we found that performing a third AFB smear for diagnostic evaluation for tuberculosis among hospitalized persons did not detect any additional cases as compared with 2 AFB smears. We also found that the overall sensitivity to detect tuberculosis was higher in any diagnostic algorithm involving 1 or 2 NAATs (74%–79%) when compared with 3 smears alone (60%). As expected, NAATs were more often positive when performed on AFB smear–positive specimens than negative (100% vs 62%, *P* < .01). In 9% of individuals, sputum could not be produced, and bronchoscopic sampling was pursued for diagnosis.

The observed low yield of performing a third AFB smear as recommended by guidelines of the Centers for Disease Control and Prevention raises the question of whether 2 AFB smears, particularly when used in conjunction with 1 or more NAATs, would be sufficient among people undergoing evaluation for tuberculosis in a low-incidence setting. In our setting, sputum induction is readily available, which may have led to a higher yield on the first 2 smears and, therefore, a lower incremental yield on a third smear. Many of our study sites routinely concentrate specimens and use fluorochrome staining (supplementary appendix), and each practice has been shown to increase the sensitivity of smears for detection of *M tuberculosis* by approximately 10% [7, 18, 19]. A third AFB smear may provide a higher incremental yield in settings where these strategies are not routine.

Our findings regarding the low yield of a third smear in the assessment for pulmonary tuberculosis are consistent with a systematic review, which found that the incremental yield of a third smear was only 2.3% [20]. A recent study from the Mayo Clinic concluded that collecting >2 respiratory specimens did not lead to any additional tuberculosis diagnoses [21]. Collecting a third specimen contributes to patient time in airborne isolation, attendant bed capacity challenges, and strain on hospital laboratory resources. These considerations, taken with our results, support routine use of 2 rather than 3 AFB smears for routine tuberculosis diagnosis in low-prevalence settings, particularly when a NAAT is available. However, given that a small proportion of participants in our study (3%) required 3 specimens to yield a positive mycobacterial culture, collecting a third for smear and culture may still be indicated in select circumstances when the pretest probability for tuberculosis remains high after 2 negative samples.

We found that using 1 or more NAATs substantially increased the sensitivity of any tuberculosis diagnostic algorithm. A recent systematic review and meta-analysis revealed that among people with suspected tuberculosis and positive respiratory cultures for *M tuberculosis*, the sensitivity of detecting tuberculosis with 1 NAAT is 86% (95% CI, 75%–92%) and even higher with 2 NAATs at 92% (95% CI, 84%–96%) [22]. These estimates are moderately higher than what we observed, potentially related to our inclusion of individuals who were not able to produce sputum, suggesting paucibacillary disease [23], and instead required diagnosis on bronchoscopic specimens.

Despite the higher sensitivity of NAATs over AFB smears, NAATs are still underutilized, largely due to concerns regarding cost [16], which limit their availability in health care facility–based laboratories. NAATs can detect *M tuberculosis* within hours, much earlier than mycobacterial cultures, which usually require 2 to 8 weeks to grow [6, 24]. In a study in North Carolina, use of NAATs reduced airborne isolation duration from 68.0 hours with AFB smears alone to 20.8 and 41.2 hours with 1- and 2-NAAT approaches, respectively [12]. In a retrospective study in New York, use of a NAAT resulted in an estimated cost savings of $4445 to $5947 per patient as compared with conventional AFB smear–based testing due to decreased hospital length of stay [4]. Given these findings and those of our study, health care facilities should consider expanded use of NAATs to reduce facility length of stay, laboratory resource use, and delays in treatment initiation. However, AFB smears and mycobacterial cultures remain critical components of any tuberculosis diagnostic algorithm, as they help guide exposure investigations and allow for drug susceptibility testing to inform treatment decisions.

It was notable that 28% of individuals in our cohort with culture-positive tuberculosis were not recognized by AFB smear or NAAT. Some of these had only 1 or 2 specimens collected for diagnostic evaluation, either because they could not provide sufficient sputum specimens or because a nonclassical presentation may have led to incomplete evaluation for tuberculosis. Premature discontinuation of transmission-based precautions carries risk of nosocomial transmission and labor-intensive exposure investigations [2]. A single-hospital study in New York City found that each case of unrecognized tuberculosis resulted in a mean of 41 exposed staff and an average of 38 person-hours per contact investigation [3]. Our study highlights the importance of using clinical judgment prior to discontinuation of airborne precautions, even if AFB smear and NAAT results are negative.

Similar to other published studies, we found that the observed yield of specimens obtained from sputum and tracheal aspirates was higher than that of specimens obtained through bronchoscopy [25–29]. It is likely that mycobacterial burden was higher among participants who were able to produce sputum than among those who may have required bronchoscopy, contributing to this observed difference [23]. NAATs, however, require additional laboratory validation to be used on bronchoscopic specimens since they are not a Food and Drug Administration–approved specimen type for NAATs (supplementary appendix), which could contribute to this difference as well. In our study, 13% of participants were diagnosed with pulmonary tuberculosis through bronchoscopy, either because they could not produce sputum or because tuberculosis was not suspected at the time of bronchoscopy. This finding supports current guidelines that bronchoscopy should still be considered if sputum induction is unsuccessful or if miliary tuberculosis is suspected and other sputum studies are negative [7].

Our study has several important limitations. First, in this retrospective study, some participants underwent only 1 or 2 AFB smears and cultures and/or no NAATs as part of their evaluation. It is possible that additional individuals with only 1 or 2 negative cultures performed would have been diagnosed if more cultures had been performed. However, this approach allowed for the evaluation of diagnostic algorithm performance among patients for whom the pretest probability for tuberculosis may have been moderate or low and those who could not produce sputum. Second, our cohort size limited our ability to assess for possible contributors to the observed differences in sensitivity between sputum or tracheal aspirates and bronchoscopic specimens; it is likely that the lower sensitivity observed with bronchoscopic specimens is due in part to paucibacillary disease in those who were less able to produce sputum. Last, we assessed the sensitivity of smears and NAATs performed on respiratory specimens collected within a 7-day window from hospitalized persons in a low-prevalence setting. Mycobacterial diagnostic algorithm characteristics may be different in high-prevalence settings, among ambulatory persons with earlier disease, or when multiple specimens are collected over longer intervals.

In conclusion, our findings support reduced use of third AFB smears in favor of increased prioritization of NAATs in the evaluation of suspected pulmonary tuberculosis in low-prevalence settings, although a third smear and culture could be considered when pretest probability for tuberculosis is high. Such a strategy should be considered in future updates to national guidelines on the evaluation of persons with suspected pulmonary tuberculosis in low-prevalence settings.

## Supplementary Data

Supplementary materials are available at *Open Forum Infectious Diseases* online. Consisting of data provided by the authors to benefit the reader, the posted materials are not copyedited and are the sole responsibility of the authors, so questions or comments should be addressed to the corresponding author.

## Supplementary Material

ofae253_Supplementary_Data

## Notes

***Acknowledgments.*** We thank Jasmine B. Ha, MS, Mass General Brigham Digital Health, for project management support, as well as Katie Swanson, RN, and Lauren West, MPH, for data analysis support. We also acknowledge Meghan Baker, MD, and Matthew Leibowitz, MD, for providing study site–specific information about tuberculosis diagnostic algorithms and procedures at facilities across the Mass General Brigham network.

***Author contributions.*** C. M. D.: conceptualization, methodology, validation, formal analysis, investigation, data curation, writing–original draft, visualization. K. C. Z.: conceptualization, methodology, supervision. D. S. M.: software. J. A. B., S. E. T.: methodology. C. V. G.: Project administration. R. C., A. D., E. G., and M. S. J.: investigation, data curation, validation. E. S. S.: conceptualization, methodology, supervision, resources, funding acquisition. All authors: writing–review and editing.

***Patient consent statement.*** No patient consent was required for this study. The study was approved by the Mass General Brigham Institutional Review Board (2012P002359).

***Financial support.*** This work was supported by Centers for Disease Control and Prevention (NU50CK000622 to E. S. S.); the Massachusetts General Hospital Executive Committee on Research (to C. M. D.); and the Eunice Kennedy Shriver National Institute of Child Health and Human Development (K08HD101342 to C. M. D.).
