# Supplementary material for: Second Time's the Charm? Assessing the Sensitivity and Yield of Inpatient Diagnostic Algorithms for Pulmonary Tuberculosis in a Low-Prevalence Setting
Source: Open Forum Infect Dis. 2024 May 3;11(6):ofae253. doi: 10.1093/ofid/ofae253 (PMC11170497; doi:10.1093/ofid/ofae253)
Supplement: ofae253_Supplementary_Data [file ofae253_supplementary_data.pdf]

Second time's the charm? Assessing the sensitivity of inpatient testing algorithms for pulmonary tuberculosis in a low-prevalence setting

Supplemental Appendix

Caitlin M. Dugdale, MD *et al.*

Study sites and laboratory procedures across the Mass General Brigham (MGB) Healthcare System

Data regarding the results of mycobacterial cultures, acid-fast bacilli (AFB) smears, and nucleic acid amplification tests (NAATs) were available from eight Mass General Brigham acute care facilities during the study period, including: Brigham and Women's Faulkner Hospital (BWF), Brigham and Women's Hospital (BWH), Cooley Dickinson Hospital (CDH), Massachusetts General Hospital (MGH), Martha's Vineyard Hospital (MVH), Nantucket Cottage Hospital (NCH), Newton Wellesley Hospital (NWH), and Salem Hospital (SLM). During the study period, each facility had a site-specific approach to the evaluation of pulmonary tuberculosis among hospitalized persons based on available hospital and laboratory resources. Most facilities required either 1) three negative AFB smears collected at least eight hours apart +/- one NAAT or 2) two negative AFB smears collected at least eight hours apart and two negative NAATs in order to discontinue airborne isolation in the setting of suspected tuberculosis. In most facilities, provider attestation that tuberculosis is no longer on the differential diagnosis, or individual chart review by infection control or infectious disease providers was required to remove airborne isolation precautions as well.

Different approaches to smear microscopy and NAAT are used across MGB. During the study period, some MGB facilities performed AFB smear microscopy and the Cepheid Xpert® MTB/RIF assay (NAAT) in-house. Existing FDA approval for NAATs does not cover non-sputum respiratory specimens. Therefore, some MGB facilities that perform NAATs in-house performed additional validation exercises in order to perform NAATs on bronchial specimens; NAAT results on these specimens performed on validated platforms are included in this study. During the study period, at MGH, to differentiate between tuberculosis and NTM, it was routine practice to perform a NAAT on any specimen that had a positive AFB smear in which prior mycobacterial infection had not been diagnosed.

During the study period, not all MGB facilities had access to NAAT in their microbiology laboratory and/or they lacked the laboratory personnel to routinely perform AFB smears, and so sent respiratory specimens to the Massachusetts State Public Health Laboratory for testing to be performed. Whether AFB smear microscopy was performed on concentrated or unconcentrated specimens, and whether or not fluorochrome staining was used, also varied by MGB facility and by laboratory capacity during the study period. Studies performed on non-respiratory specimens, and those that were not completed due to having a poor quality/ contaminated specimen or quantity not sufficient for evaluation were not included in this analysis.

MGB Immunocompromised Criteria

These criteria were originally developed in the context of the COVID-19 pandemic to guide decisions regarding the duration of isolation precautions after recovery from respiratory viral infection. However, we applied these criteria to define immunocompromised status for the purpose of the chart review, as they capture a broad range of contributors to immunocompromise and have been in use throughout the MGB system since early in the COVID-19 pandemic.

1. Immunocompromised based on diagnosis. Persons with any of the following diagnoses will be considered immunocompromised for the purposes of this policy.

a) HIV CD4 <200 cells/mm^3
b) Active lymphoma or leukemia (including indolent CLL)
c) Metastatic cancer, not in durable remission
d) Cytotoxic chemotherapy within the prior 3 months
e) Radiation therapy within prior 3 months
f) Congenital immunodeficiency
g) Aplastic anemia
h) Solid organ transplant recipients on immunosuppressive therapy
i) Hematopoietic stem cell transplant recipients, unless >2 years post-transplant AND no longer on any immunosuppressive therapy

2. Immunocompromised based on medications. Persons with receipt of the following medications will be considered immunocompromised for the purposes of this policy.

a) Glucocorticoid therapy: the equivalent of prednisone 20 mg/d or more, for 2 weeks or more, or if such therapy has been discontinued within the past month
b) Alkylating agents (e.g. cyclophosphamide) within the past 3 months
c) Antimetabolites: methotrexate >0.4 mg/kg/week, azathioprine >3 mg/kg/day, 6- MP >1.5 mg/kg/day) within the past 3 months
d) Cyclosporine, tacrolimus, sirolimus, everolimus, mycophenolate mofetil (usually given in the context of organ transplant, but sometimes used for other indications) within the past 3 months
e) Biologic immunosuppressants and immunomodulators (not including immune checkpoint inhibitors) within the past 3 months (6 months for lymphocyte-depleting agents)

Growth of non-tuberculous mycobacteria (NTM) in cultures

We evaluated the growth of NTM in mycobacterial cultures obtained during the testing episode in which culture-confirmed pulmonary tuberculosis was diagnosed. A "testing episode" was defined as the window within 7 days of the initial specimen in a series being collected with any specimen in that series eventually having a mycobacterial culture positive for *Mycobacterium tuberculosis*. Of the 104 participants with culture-confirmed pulmonary tuberculosis, 11 (11%) demonstrated growth of NTM in one or more of the mycobacterial cultures obtained from the TB testing episode (Appendix Table 2).

Appendix Table 1. Characteristics of participants with pulmonary tuberculosis diagnosed on the third mycobacterial culture

History at presentation	Radiographic findings	Specimen type of positive culture	Third AFB smear result	NAAT result
86yo female from a TB endemic country with PMH of diabetes and Stage IV chronic kidney disease who presented with 3 weeks of progressive shortness of breath and cough and only mild improvement with treatment for presumed community acquired pneumonia	CT chest with soft tissue consolidation in the superhilar region extending to the inferior right upper lobe with associated partially calcified lymphadenopathy and bilateral pleural effusions.	Sputum/tracheal aspirate	Negative	Not performed
76yo female from a TB endemic country with PMH of a positive T-spot without prior LTBI treatment who presented with palpitations, lightheadedness, shortness of breath, and syncope.	CT chest with lobulated, irregular, peribronchial, tree-in-bud opacities in the bilateral upper lobes with mild biapical scarring without associated lymphadenopathy.	BAL	Negative	Negative (from BAL)
80yo female from a TB endemic country with PMH of a laryngeal mass noted as an outpatient who presented with a several month history of weight loss, coughing after eating, and right-sided throat discomfort. Underwent fiberoptic exam with biopsy of laryngeal lesion that demonstrated necrotizing granulomas, raising concern for tuberculosis.	CT chest with biapical nodularity/scarring, diffuse mosaic attenuation, multiple scattered nodules, and tree-in-bud opacities in the right middle and right lower lobes.	Sputum/tracheal aspirate	Negative	Negative (from sputum)

AFB: Acid-fast bacilli, NAAT: nucleic acid amplification test, PMH: past medical history, CT: computed tomography, BAL: bronchoalveolar lavage.

Appendix Table 2. Growth of non-tuberculosis mycobacteria in respiratory cultures obtained during the TB testing episode

Participant #	NTM growth in culture	# mycobacterial cultures obtained	# cultures with NTM growth	# cultures with MTB growth
1	Mycobacterium abscessus	4	1	2
2	Mycobacterium abscessus	2	2	2
3	Mycobacterium avium intracellulare complex	3	1	1
4	Mycobacterium chimaera intracellulare group	3	2	2
5	Mycobacterium chimaera intracellulare group	3	1	1
6	Mycobacterium chimaera intracellulare group	3	1	3
7	Mycobacterium fortuitum	2	1	2
8	Mycobacterium gordonae	3	1	2
9	Mycobacterium lentiflavum	2	1	1
10	Mycobacterium mucogenicum group	6	1	1
11	Mycobacterium septicum	4	2	1

Appendix Table 3. Results of sputum testing for specimens collected within 24 hours after bronchoscopy

Participant	Pre-bronchoscopy results	Bronchoscopy results	Interval between bronchoscopy and specimen collection	Post-bronchoscopy results	Diagnosis made on post-bronchoscopy sputum?
A	None	Smear negative x 1, mycobacterial culture positive x 1	<1 hour	AFB smear negative x 1, mycobacterial culture positive x 1	No
B	None	AFB smear negative x 1, NAAT negative x 1, mycobacterial culture negative x 1	24 hours	AFB smear negative x 1, NAAT positive x 1, mycobacterial culture positive x 1	Yes
C	1 / 2 AFB smears positive, 2 /2 cultures positive	Smear positive x 1, NAAT positive x 1, culture positive x 1	22 hours	Smear positive x 1, culture positive x 1	No
D	None	Smear positive x 1, NAAT positive x 1, culture positive x 1	2 hours	Smear negative x 1, culture positive x 1	No
E	Smear negative x 1, culture negative x 1	Smear positive x 1, NAAT positive x 1, culture positive x 1	2 hours	Smear positive x 1, culture positive x 1	No
F	None	Smear negative x 1, NAAT negative x 1, culture positive x 1	20 hours	Smear negative x 1, culture negative x 1	No
G	Smear negative x 1, culture positive x 1	Smear negative x 1, NAAT negative x 1, culture positive	<1 hour	Smear negative x 1, NAAT negative x 1, culture positive x 1	No
H	Smear negative x 1, culture positive x 1 / 2	Smear negative x 2, NAAT negative x 1, culture negative x 2	9 hours	Smear negative x 1, culture negative x 1	No
